# The Assessment of Race, the 21-Gene Recurrence Score, and Breast Cancer Outcomes at Kaiser Permanente

**DOI:** 10.3390/cancers18162662

**Published:** 2026-08-18

**Authors:** Amanda F. Petrik, Charisma L. Jenkins, Ana G. Rosales, Terry Kimes, Ning Smith, Nathalie Johnson, Amy Morris, Suma Vupputuri, A. Blythe Ryerson, Matthew P. Banegas, Robert B. Hufnagel, Lilian G. Perez, David Mosen

**Affiliations:** 1 Kaiser Permanente Center for Health Research, Portland, OR 97227, USA; charisma.l.jenkins@kpchr.org (C.L.J.); ana.g.rosales@kpchr.org (A.G.R.); terry.kimes@kpchr.org (T.K.); ning.x.smith@kpchr.org (N.S.); david.m.mosen@kpchr.org (D.M.); 2 Legacy Health, Portland, OR 97210, USA; njohnson@lhs.org; 3 Kaiser Permanente Northwest, Portland, OR 97227, USA; almorr503@gmail.com; 4 Mid-Atlantic Permanente Research Institute, Kaiser Permanente Mid-Atlantic States, Washington, DC 20002, USA; suma.vupputuri@kp.org (S.V.); lilian.g.perez@kp.org (L.G.P.); 5 Center for Research and Evaluation, Kaiser Permanente Georgia, Atlanta, GA 30305, USA; blythe.ryerson@kp.org; 6 Moore’s Cancer Center, University of California San Diego, San Diego, CA 92037, USA; mbanegas@health.ucsd.edu; 7 Center for Integrated Health, Honolulu, HI 96819, USA; robert.b.hufnagel@kp.org

**Keywords:** breast cancer disparities, Oncotype DX, Black and African American patients, recurrence score, mortality, treatment outcomes, cancer prognosis, health equity

## Abstract

**Background**: It is well documented that Black and African American (AA) patients have the highest risk of recurrence of breast cancer. However, less is known about racial differences in gene expression profiling tests such as Oncotype DX that predicts cancer recurrence. This study aimed to assess the predictive differences in Oncotype DX scores for breast cancer outcomes between Black/AA and non-Hispanic White patients. **Methods**: We identified Oncotype DX testing status in patients ages 18–75 years who were newly diagnosed with breast cancer, assessed race and other factors among patients with different testing statuses, and described the distribution of the Oncotype DX score. Additionally, we assessed treatment, and multifactorial impact on outcomes by Oncotype DX score, including: receipt of chemotherapy, endocrine therapy, stage, and disease-related recurrence and mortality. **Results**: We identified 2577 eligible patients for the analysis. Black/AA patients had a 49% greater risk/hazard of mortality than non-Hispanic White (NHW) patients (multivariable model *p*-value 0.0444). Oncotype DX score differences were associated with mortality in the overall sample (multivariable model, *p*-value = 0.0281). Specifically, in the Oncotype DX-specific models, Black/AA patients with an Oncotype DX score of 0–16 had 73% greater risk of mortality than NHW patients (multivariable model *p*-value 0.0409). While all other Oncotype DX score categories showed elevated risks of mortality, they were not statistically significant. The Oncotype DX-specific multivariable models showed that, compared to NHW patients, recurrence was lower for Black/AA patients in the lower categories and higher in the upper categories, but results were not statistically significant. **Conclusions**: This study assessed recurrence and mortality outcomes by the recurrence score and found significantly higher rates of mortality among Black/AA patients in the lowest recurrence score category. Other studies have shown that recurrence scores add prognostic value during diagnosis. However, examining performance of these tools among diverse populations is imperative.

## Simple Summary

This study looks at whether a common breast cancer risk test gives equally useful information for Black and African American patients as it does for White patients. The authors want to see how test scores relate to cancer recurrence and survival, and whether the test works the same across different groups. The findings suggest that Black patients may still face higher death risk even when their test score is lower, which means more research is needed to make sure these tools support fair and accurate care for everyone.

## 1. Introduction

There are persistent disparities in cancer outcomes among diverse groups of patients. Black and African American (AA) patients have the highest risk of recurrence of breast cancer [1]. Additionally, survival rates are lower for non-Hispanic Black patients (87.1%) compared to any other racial/ethnic group of patients with similar hormone receptor–positive/HER2-negative, lymph node–positive breast cancer (non-Hispanic White: 91.6%, Asian: 93.9%, and Hispanic: 91.4%) [2]. Consequently, tools that enable the identification of patients at greatest risk for recurrence may help inform care to improve outcomes among Black and African American (AA) patients with breast cancer.

The Oncotype DX recurrence score assesses the expression of 21 genes in a tumor sample using reverse transcription polymerase chain reaction to estimate the risk of recurrence [3]. It has been used, in combination with clinical context (i.e., age, nodal status, menopausal status, treatment preference), to assess patients for low, intermediate, or high risk of negative outcomes (mortality and recurrence). Scores range from 0 to 100, where a low-risk score (0–16) indicates that a patient has a low chance that the cancer will recur within 5 years, while a high-risk result (26–100) suggests that a patient has a higher chance that their cancer will recur within 5 years [4]. Scores indicate if the benefits of chemotherapy are likely to be greater than the side effects. Studies have shown racial differences in the predictive value of recurrence scores like Oncotype DX [5,6]. The 21-gene Oncotype DX recurrence score assay was validated in studies that enrolled predominantly White patients, with far less representation of non-White patients [5]. The score plays a primary role in chemotherapy recommendations. Furthermore, evidence suggests there may be disparities in using these scores, especially among Black/AA patients, largely due to access and financial barriers [7]; therefore, the performance of Oncotype DX in racial minority individuals deserves further investigation [8].

This project closely examines the epidemiology of breast cancer and the Oncotype DX scores among Black/AA and White patients enrolled in three separate Kaiser Permanente (KP) health system regions (Northwest, Georgia, and Mid-Atlantic). Using data from a cohort of breast cancer patients, the objectives of the study were to: (1) assess cumulative survival by race and Oncotype DX score; (2) assess the cumulative incidence of recurrence by race and Oncotype DX score; (3) compare the risk of death and recurrence between Black/AA and White patients; and (4) compare the risk of death and recurrence for Black/AA and White patients by Oncotype DX score.

## 2. Methods

### 2.1. Study Design and Setting

This was a retrospective cohort study using cancer registry and electronic health record data from three Kaiser Permanente regions. The analytic dataset was derived from the cancer registries at each of the three participating KP health systems. KP is an integrated, membership-based health system that operates across seven regions and combines insurance coverage with coordinated care delivery. Members receive care across inpatient and outpatient settings, along with pharmacy, laboratory, imaging, and other support services, all connected through a shared electronic health record (EHR). KP Mid-Atlantic States serves about 750,000 members in Washington, DC, Maryland, and northern Virginia. KP Northwest serves approximately 630,000 members in Oregon and southwest Washington, and KP Georgia serves around 320,000 members in the Atlanta metropolitan area. Each region maintains its own cancer registry, where trained registrars systematically collect and document data for all reportable cancer cases.

### 2.2. Study Population

The population included non-Hispanic White (NHW) and Black/AA patients who were aged 18–75 years, biologically female at birth, and diagnosed with primary invasive estrogen-positive and HER-2-negative breast cancer with surgical evaluation from 2010 to 2022. To be eligible for the cohort, patients had to have no prior cancer diagnosis, be enrolled as a KP member for at least 1 year prior to diagnosis, have an Oncotype DX score, and not be on internal research exclusion lists. Eligible patients were followed for 5.3 years on average following their biopsy date.

### 2.3. Measures

We obtained electronic health record data on patient characteristics, including tumor characteristics, comorbidities, and Oncotype DX scores. The main exposures of interest were patients’ Black/AA or NHW race and Oncotype DX categorized into 4 groups based on literature and distribution: 0–16 (low risk of recurrence), 17–20 (medium–low risk of recurrence), 21–25 (medium–high risk or recurrence), and 26–100 (high risk of recurrence). Race and ethnicity were based on EHR and tumor registry data, with the hierarchy of Black/AA, Hispanic, NHW, other, and unknown. We also assessed the following covariates: demographic characteristics including neighborhood deprivation index (NDI) and rurality, body mass index (BMI), tumor size, node positivity, surgery type, stage of cancer (SEER Summary Stage), whether chemotherapy was offered, health insurance characteristics, menopausal status (based on age > 55), Charlson comorbidity score, and Oncotype DX score.

### 2.4. Outcomes

The outcomes included cancer recurrence and mortality. Death was ascertained by both the EHR and death registries, and cause of death was not assessed. Cancer recurrence was ascertained from data from the cancer registries. Recurrence was defined from an indicator in the tumor registry, or 2 subsequent diagnoses within 6 months of each other after the primary diagnoses as a proxy for recurrence. Outcomes were reported by receipt of the Oncotype DX score and by the Oncotype DX risk score group typical score ranges.

### 2.5. Statistical Analysis

All patient characteristics were reported as means (and standard deviations) or frequencies (and percents) by race: Black/AA versus non-Hispanic White. Categorical variable comparisons were assessed using Chi-square tests while continuous variable comparisons were evaluated by Wilcoxon Rank-Sum tests.

We used univariable and multivariable Cox proportional hazard models to examine if disease recurrence and time to disease recurrence differed among race groups and among Oncotype DX Risk score levels. Death was considered as a competing risk in all recurrence models. We first ran univariable models of demographic, tumor, and treatment variables.

A variable was included in the multivariable model if it was statistically significant at the 0.10 level in the univariable model. However, we chose a priori a set of predefined candidate independent variables that were likely confounders based on expert opinion as well as the extant literature. These variables were evaluated empirically, and all proved to be statistically significant at 0.10. We assessed multicollinearity using the variance inflation factor. Gray tests were employed when assessing significant differences among the cumulative risk of recurrence functions. We also ran univariable and multivariable models in each of the Oncotype DX risk score groups. The only variable of interest in these stratified models was race. The multivariable models were adjusted for the most statistically relevant variables. Cumulative risk of recurrence functions was also assessed by Gray test in each of the Oncotype DX score strata.

Mortality models were estimated in a similar fashion as recurrence models except that recurrence in the mortality models was not treated as competing risk. Thus, the cumulative risk of death functions was evaluated by the regular log-rank test.

## 3. Results

**Figure 1:**
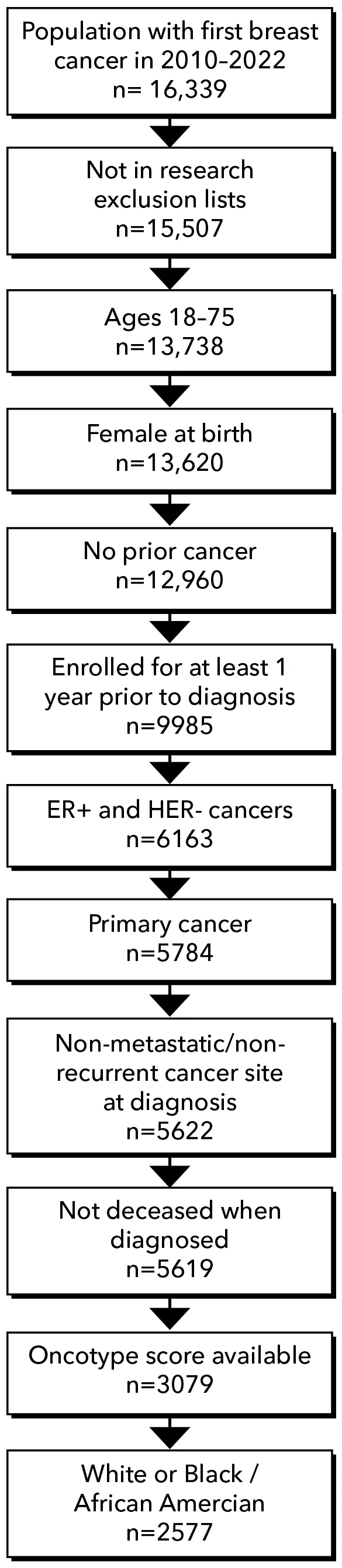
CONSORT.

We identified 2577 eligible patients for the analysis, including 766 Black/AA and 1811 White patients. Across all three regions, 16,339 patients were identified with a first breast cancer in 2010–2022. Patients were included if they were not on research exclusion lists, 18–75 at diagnosis, biologically female at birth, did not have a prior cancer, or had been enrolled in KP for at least a year (Figure 1). We included patients with a diagnosis of invasive estrogen-positive and HER-2-negative breast cancer and if it was the primary cancer. We also included patients with a recurrent or metastatic site prior to diagnosis, who did not die prior to diagnosis, who did not have a missing Oncotype DX score, or who were not White or Black/African American. Patients who were missing the Oncotype DX score were older, diagnosed more recently, more likely to have positive nodes, larger tumors, higher cancer stage, a mastectomy, and have received chemotherapy more often than patients with the Oncotype DX score (Supplementary Table S1).

Patient characteristics are detailed in Table 1. Compared to NHW patients, Black/AA patients were younger at diagnosis (78.1% vs. 83.2% > 51 years of age, *p* = 0.0023), more often had mastectomies (30.3% vs. 27.1%, *p* = 0.0003), more often had received chemotherapy (22.1% vs. 15.4%, *p* < 0.0001) and more often had higher Oncotype DX score categories (21–25 = 16.4% and 26–100 = 21.7% for Black/AA patients vs. 11.6% and 12.8% for NHW patients, *p* < 0.0001). Stage of cancer was not different between Black/AA and NHW patients. BMI was higher in Black/AA patients than in NHW patients (32.9 vs. 30.1, *p* < 0.0001). Endocrine therapy was received more often in Black/AA women than in NHW women (91.1% vs. 88.4%, *p* < 0.0420).

### 3.1. Mortality

**Figure 2:**
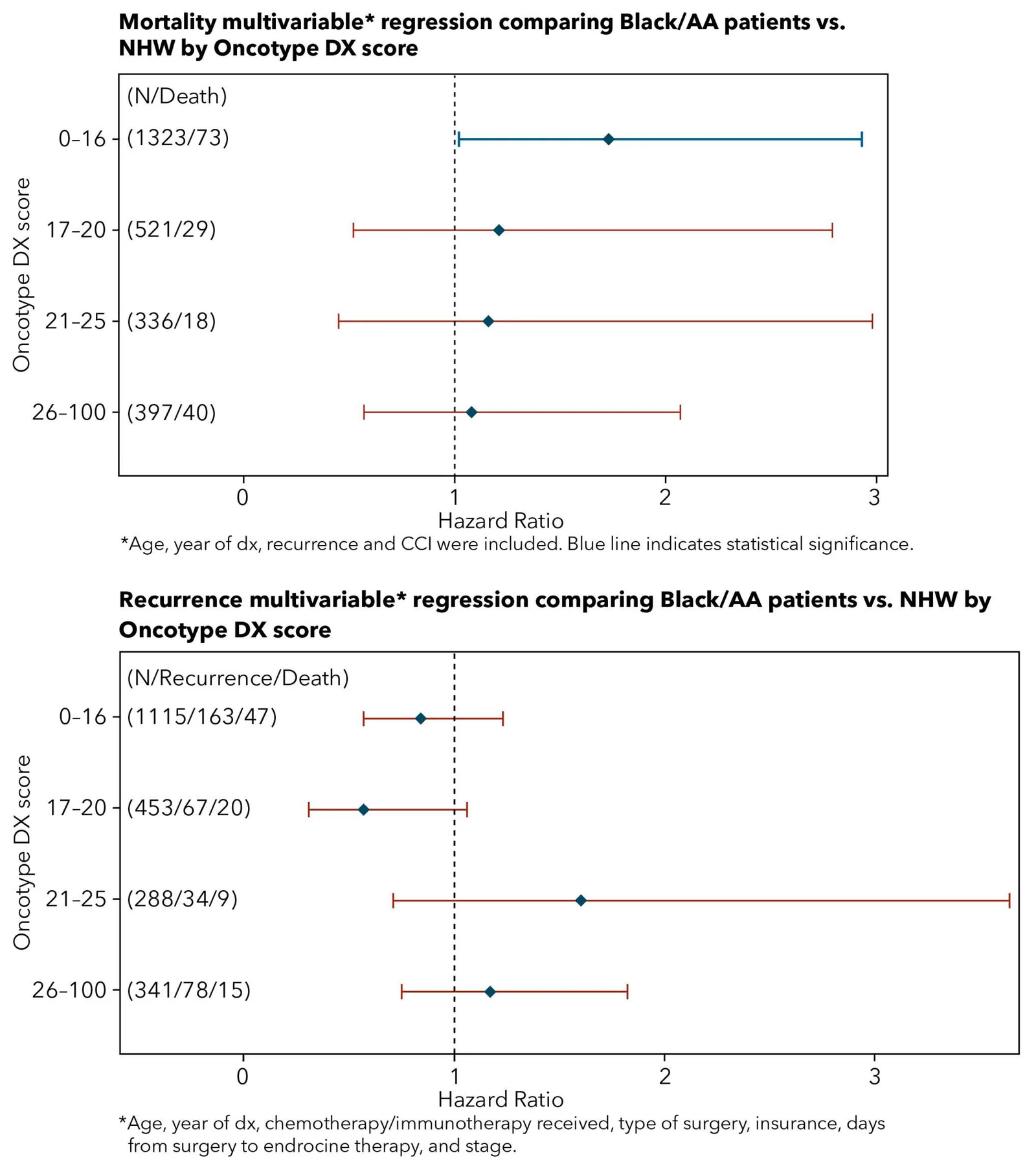
Mortality and recurrence comparing Black/AA to NHW patients by Oncotype DX score (estimated hazard ratios with 95% CI).

The analysis of race and mortality by Oncotype DX assessed 2577 patients with breast cancer. Patients (*n* = 316) with incomplete covariate data were excluded from this analysis due to missing stage, NDI, and time from surgery to endocrine therapy. Figure 2 shows the mortality regression and hazard ratios for Black/AA patients to NHW patients stratified by Oncotype DX category (0–16, 17–20, 21–25 and 26–100). All models exhibit elevated mortality among Black/AA patients compared to NHW; only the lowest Oncotype DX category is significant.

The results from a Cox proportional hazard analysis are presented in Figure 2 (full model in Supplementary Table S2). The multivariable model (Table 2) included age, year at diagnosis, age at menopause, stage, chemotherapy/immunotherapy received, days of surgery to endocrine therapy, recurrence, the Charlson comorbidity index, insurance, neighborhood deprivation index, race, and Oncotype DX score. In our cohort of women with breast cancer, variables significantly associated with an increased risk of death included cancer diagnosis in 2010–2017 (vs. 2018–2022), stage 2 or 3 cancer (vs. stage 0 or 1), higher number of days to endocrine therapy, breast cancer recurrence, Charlson comorbidity score of 1–2 or 3+ (vs. 0), Medicaid or Medicare insurance, Black/AA race, or Oncotype DX score of 26–100. In our cohort, Black/AA patients had a 49% greater risk/hazard of mortality than NHW patients (multivariable model, *p*-value 0.0444). Oncotype DX score differences were significantly associated with mortality (multivariable model *p*-value = 0.0281, Table 2). Furthermore, in the Oncotype DX-specific models (Figure 2) controlling for age, year of diagnosis, recurrence and the Charlson comorbidity score, Black/AA patients with an Oncotype DX score of 0–16 had 73% greater risk of mortality than NHW patients (*p*-value 0.0409). While all other Oncotype DX score categories showed elevated risks of mortality for Black/AA patients compared to NHW patients, they were not statistically significant.

### 3.2. Recurrence

Our analysis of recurrence by Oncotype DX score assessed 2492 patients with breast cancer. Patients (*n* = 85) who never were disease free were excluded from this analysis. Figure 2 shows the recurrence regression comparing Black/AA patients to NHW patients stratified by Oncotype DX category (0–16, 17–20, 21–25 and 26–100). This figure shows the hazard ratios for Black/AA patients compared to NHW patients; no category was statistically significant. The results from the competing risk analysis are presented in Table 3 and Figure 2 (and Supplementary Table S3). The multivariable model included age at diagnosis, year of diagnosis, treatment, stage, type of surgery, insurance, race and Oncotype DX score. The Oncotype DX-specific multivariable models (Figure 2) showed that, compared to NHW patients, recurrence was lower for Black/AA patients in the 0–16 and 17–20 Oncotype DX categories and higher in the 20–25 and 26–100 categories, but the results were not statistically significant (Patient Population by Black/African American Race limited to 0–16 Oncotype Dx score are presented in Supplementary Table S4).

## 4. Discussion

In this study among adult patients with newly diagnosed breast cancer who had an Oncotype DX recurrence score, we found significantly higher risk of mortality among Black/AA patients in the lowest recurrence score category, but no statistical difference in recurrence of cancer. These findings should be interpreted in the context of notable data limitations in recurrence data described in the limitations section that may have reduced our ability to detect true differences in recurrence outcomes.

A higher Oncotype DX score is an indicator of higher likelihood of recurrence and greater benefit from chemotherapy [3]. The Oncotype DX score has been found to be strongly associated with death [9]. Many studies have shown that recurrence scores add prognostic value during diagnosis [10]. However, examining the performance of these tools among diverse populations is imperative.

Differential outcomes in Black/AA patients compared to NHW patients have historically been attributed to failure to adhere to recommended surveillance and treatment [11,12]. Press and colleagues suggested that racial equality in use of chemotherapy would be improved if there was increased access to Oncotype DX testing; they found greater omissions of Oncotype DX testing in Black/AA patients but no differences in chemotherapy compared to NHW [13]. In our study, we found that, despite similar stages of cancer, Black/AA patients more frequently presented with higher Oncotype DX scores. These support results from Hoskins et al. who found that Black/AA patients were significantly more likely than NHW women to have a score greater than 25 even when controlling for age, year of diagnosis, tumor size, and nodal status [14].

The goal of genomic testing is to be prognostic and predictive of therapy benefit, and it is being used more widely to guide treatment recommendations. It is imperative that testing be agnostic to racial diversity or adjusted or recalibrated to account for differences. This will help to correct disparate outcomes. Across studies evaluating genomic risk stratification in racially diverse breast cancer populations, MammaPrint and Oncotype DX show different patterns in how they classify tumors from Black/African American patients. Evidence from the FLEX and BEST registries [15] shows that MammaPrint identifies a significantly higher proportion of Black/AA women as “high risk” compared with White women, suggesting that MammaPrint may be more sensitive to aggressive tumor biology that disproportionately affects Black patients. In contrast, multiple analyses of Oncotype DX in hormone receptor positive breast cancer patients show that Black/AA patients often receive lower Oncotype DX recurrence scores than would be expected based on their higher observed recurrence and mortality rates, raising concerns that Oncotype DX may underestimate mortality risk in the Black/AA population [16]. These important differences in how these assays capture race-associated tumor biology underscore the need for diverse validation study cohorts.

The performance of recurrence scores when accounting for surveillance and treatment adherence has yet to be explored; however, Ibraheem identified performance issues with recurrence scores across racial and ethnic groups, with better performance for White patients [8]. Jung and colleagues also found that the predictive value of the Oncotype DX score was “not warranted” among non-White patients [5]. Oncotype DX testing often is only done on early-stage tumors, and on patients who have breast surgeries, indicating that use of recurrence scores may be streamlined.

The overall performance of recurrence scores among Black/AA patients needs serious consideration in breast oncology care as it is a significant component of treatment planning and may be impacting outcomes in Black/AA women in the lowest risk quartile. Improving accuracy of risk prediction would facilitate treatment choices that can improve disease outcomes. Diligent review of testing options or exploration of tumor endocrine sensitivity for Black/AA patients in the low-risk group should be considered upon initial breast cancer diagnosis. The WSG-ADAPT trial and POETIC study both studied short courses of presurgical endocrine therapy as an option to confirm endocrine sensitivity [17,18]. This is seen as static (genomic score) and dynamic (response to therapy) evaluation which then confirms the choice of endocrine therapy only or informs the decision to add chemotherapy even with lower genomic score if the tumor is not endocrine-sensitive. Tailored treatment recommendations such as these should be considered for Black/AA breast cancer patients.

Finally, developing an improved risk assessment tool for newly diagnosed breast cancer patients, in combination with a shared decision-making tool, could be considered. An improved risk assessment tool can improve longer-term outcomes for Black/AA patients diagnosed with breast cancer, and future research could examine a newly revised Oncotype DX score on oncology related outcomes for Black women. Minimally, a more thorough investigation of the performance of low-scoring Black/AA patients should be conducted.

## 5. Limitations

This study is limited in that the assessment was only conducted among Kaiser Permanente patients. Further assessments should be conducted with patients in non-integrated health systems. This study is also limited in that there were not enough patients to conduct an analysis among other groups of patients like Hispanics; further analysis should assess outcomes among diverse patients. It is worth noting that racial differences were not significant for the recurrence outcome. A persistent issue in US data is that patients change health systems and recurrence data may not be available. Further, there were missing data issues due to local registry abstraction patterns, such as incomplete case abstractions for earlier study period years, as well as differences in local registry reporting requirements that may not capture cancer recurrence information accurately.

## 6. Conclusions

Among patients in this integrated health system cohort with available Oncotype DX testing, Black/African American race was associated with higher all-cause mortality, including in the lowest recurrence score stratum. However, race-by-Oncotype DX interaction tests were not statistically significant, recurrence differences were not statistically significant, and further validation in diverse populations is needed. Compared with non-Hispanic White patients, Black and African American patients were somewhat younger at diagnosis, more often had mastectomy and chemotherapy, and were more likely to fall into higher Oncotype DX score categories, while cancer stage was similar between groups. Consistent with previous literature, Black and African American patients had a 49% higher risk of death than non-Hispanic White patients overall, and a 73% higher risk of death in the lowest Oncotype DX category.

The Oncotype DX breast cancer recurrence scores may not work equally well for Black and African American patients as for non-Hispanic White patients for predicting mortality. Black and African American patients often have worse outcomes even when their scores are low, which raises concern that the test may underestimate risk in this group. Future research should assess if additional testing and more personalized treatment decisions are needed for Black and African American patients to reduce breast cancer mortality and improve outcomes.

## Figures and Tables

**Table 1:** Patient population characteristics by race.

	All		Race				*p*-Value
			NHW		Black/AA		
	*N* = 2577		*N* = 1811		*N* = 766		
	*N*	COL%	*N*	COL%	*N*	COL%	
**Age at dx ≥ 51**							0.0023
No	473	18.4	305	16.8	168	21.9	
Yes	2104	81.6	1506	83.2	598	78.1	
**Year of dx ≥ 2017**							0.0009
No	828	32.1	618	34.1	210	27.4	
Yes	1749	67.9	1193	65.9	556	72.6	
**Has positive nodes**							0.7272
Missing	29	1.1	21	1.2	8	1	
No	2172	84.3	1523	84.1	649	84.7	
Yes	376	14.6	267	14.7	109	14.2	
**Average BMI**	*N* = 2570		*N* = 1805		*N* = 765		<0.0001
Mean ± SD	30.9 ± 7.2		30.1 ± 7.1		32.9 ± 6.9		
Median (25th, 75th)	29.8 (25.6, 35.2)		29.0 (24.9, 34.1)		32.2 (27.9, 37.0)		
**Tumor size**	*N* = 1594		*N* = 1330		*N* = 264		0.1405
Mean ± SD	18.7 ± 10.6		18.9 ± 10.6		17.9 ± 10.5		
Median (25th, 75th)	16 (12, 22)		17 (12, 23)		15 (12, 21)		
**Type of Surgery**							0.0003
Lumpectomy	1562	60.6	1088	60.1	474	61.9	
Mastectomy	723	28.1	491	27.1	232	30.3	
None	7	0.3	3	0.2	4	0.5	
Other	285	11.1	229	12.6	56	7.3	
Stage *							0.9132
Missing	3	0.1	2	0.1	1	0.1	
0, 1	2186	84.8	1538	84.9	648	84.6	
2, 3	361	14	253	14	108	14.1	
4, 5, 7	27	1	18	1	9	1.2	
**Chemotherapy/immunotherapy received**							<0.0001
No	2130	82.7	1533	84.6	597	77.9	
Yes	447	17.3	278	15.4	169	22.1	
**Endocrine therapy received**							0.042
No	278	10.8	210	11.6	68	8.9	
Yes	2299	89.2	1601	88.4	698	91.1	
**Days from surgery to endocrine therapy**	*N* = 2271		*N* = 1576		*N* = 695		0.688
Mean ± SD	108 ± 82		107 ± 75		110 ± 95		
Median (25th, 75th)	97 (50, 143)		97 (55, 139)		98 (40, 151)		
**Recurrence**							0.0163
Never disease free & no recurrence	53	2.1	29	1.6	24	3.1	
Never disease free & recurrence	32	1.2	18	1	14	1.8	
No recurrence	2100	81.5	1479	81.7	621	81.1	
Recurrence	392	15.2	285	15.7	107	14	
**Age of menopause > 55**							0.0002
No	767	29.8	500	27.6	267	34.9	
Yes	1810	70.2	1311	72.4	499	65.1	
**Charlson comorbidity index**							<0.0001
0	1722	66.8	1267	70	455	59.4	
1–2	698	27.1	462	25.5	236	30.8	
3+	157	6.1	82	4.5	75	9.8	
**Insurance**							<0.0001
Medicaid	56	2.2	26	1.4	30	3.9	
Medicare	1025	39.8	759	41.9	266	34.7	
Commercial	1446	56.1	987	54.5	459	59.9	
Private or Self	47	1.8	39	2.2	8	1	
Unknown	3	0.1			3	0.4	
**High deductible plan**							0.3829
No	2430	94.3	1703	94	727	94.9	
Yes	147	5.7	108	6	39	5.1	
**Rural**							0.0061
Missing	12	0.5	6	0.3	6	0.8	
No	2541	98.6	1782	98.4	759	99.1	
Yes	24	0.9	23	1.3	1	0.1	
**Neighborhood deprivation index ACS 2020**	*N* = 2570		*N* = 1805		*N* = 765		<0.0001
Mean ± SD	−0.26 ± 0.84		−0.44 ± 0.71		0.17 ± 0.94		
Median (25th, 75th)	−0.38 (−0.82, 0.17)		−0.49 (−0.91, −0.07)		0.04 (−0.53, 0.63)		
**Oncotype DX score**							<0.0001
0–16	1323	51.3	997	55.1	326	42.6	
17–20	521	20.2	373	20.6	148	19.3	
21–25	336	13	210	11.6	126	16.4	
26–100	397	15.4	231	12.8	166	21.7	

* SEER Summary Stage.

**Table 2:** Multivariable regression: time to death (*n* = 2577/death = 160).

Variables	Hazard Ratio	95% Confidence Limits		*p*-Value
**Age at diagnosis ≥ 51**	2.34	0.90	6.11	0.0817
**Year of diagnosis ≥ 2017**	1.94	1.23	3.04	0.0041
Stage *				
0, 1	Ref	Ref	Ref	
2, 3	0.51	0.29	0.92	0.0237
4, 5, 7	1.94	0.26	14.47	0.5200
**Chemotherapy/immunotherapy received**				
Yes vs. No	1.00	0.54	1.85	0.9890
Days from surgery to endocrine therapy *	1.00	0.99	1.00	0.0101
**Recurrence**				
Yes vs. No	4.40	2.99	6.50	<0.0001
**Age of menopause > 55**	0.89	0.44	1.79	0.7394
**Charlson comorbidity index**				
0	Ref	Ref	Ref	
1–2	1.51	1.03	2.20	0.0339
3+	3.14	1.91	5.17	<0.0001
**Insurance**				
Medicaid	3.37	1.02	11.15	0.0468
Medicare	2.53	1.61	3.97	<0.0001
Commercial	Ref	Ref	Ref	
Private or Self	0.58	0.08	4.27	0.5916
Neighborhood deprivation index (NDI) ACS 2020 *	1.09	0.88	1.34	0.4382
**Race Black/AA**	1.49	1.01	2.18	0.0444
Oncotype DX score **				
0–16	0.96	0.61	1.52	0.8715
17–20	Ref	Ref	Ref	
21–25	0.75	0.39	1.45	0.3976
26–100	1.89	1.06	3.39	0.0324

* Stage has 3 missing, NDI ACS 2020 has 7 missing and time from surgery to endocrine therapy has 306 missing. ** Oncotype DX score overall *p*-value = 0. 0281. Testing interaction, race × oncotype score interaction, *p*-value = 0.4762.

**Table 3:** Multivariable regression: breast cancer recurrence (*n* = 2492/recurrence = 392/death = 98).

Variables	Hazard Ratio	95% Confidence Limits		*p*-Value
**Age at dx ≥ 51**	1.06	0.80	1.42	0.6727
**Year of dx ≥ 2017**	1.17	0.92	1.51	0.2068
**Chemotherapy/immunotherapy received**	1.06	0.76	1.48	0.7195
Type of surgery *				
Mastectomy	1.02	0.80	1.30	0.8958
Lumpectomy	Ref	Ref	Ref	
Other	1.31	0.93	1.83	0.1210
Insurance *				
Medicaid	2.02	1.11	3.66	0.0211
Medicare	1.13	0.88	1.45	0.3357
Commercial	Ref	Ref	Ref	
Private or self	1.72	0.92	3.41	0.0920
**Race: Black/AA vs. NHW**	0.84	0.66	1.07	0.1636
**Oncotype DX score ^+^**				
0–16	0.86	0.65	1.15	0.3101
17–20	Ref	Ref	Ref	
21–25	0.95	0.63	1.44	0.8034
26–100	1.34	0.91	1.97	0.1448

* Time from surgery to endocrine therapy has 287 missing, Stage has 3 missing, type of surgery has 5 missing and insurance has 3 missing. ^+^ Testing interaction, race × Oncotype DX score interaction, *p*-value = 0.2428. Time from surgery to endocrine therapy and stage were in violation of the proportionality assumption; thus they were treated differently with a strata option.

## Data Availability

Data requests will be considered upon request by contacting the corresponding author.

## Supplementary Materials

The following supporting information can be downloaded at https://www.mdpi.com/article/10.3390/cancers18162662/s1, Table S1: Patient population with and without an Oncotype DX score; Table S2: Mortality univariate regression: all variables; Table S3: Breast cancer recurrence univariate regression: all variables; Table S4: Patient population by Black/African American race limited to 0–16 oncotype Dx score.
