# Supplementary material for: The Assessment of Race, the 21-Gene Recurrence Score, and Breast Cancer Outcomes at Kaiser Permanente
Source: Cancers (Basel). 2026 Aug 18;18(16):2662. doi: 10.3390/cancers18162662 (PMC13511634; doi:10.3390/cancers18162662)
Supplement: Supplementary file 1 [file cancers-18-02662-s001.zip › cancers-4440518-supplementary.pdf]

Supplemental Tables:

Table S1. Patient Population with and without an Oncotype DX Score.

--------------	All		Study Population: Without Oncotype DX score (N=2,524)		Study Population: With Oncotype DX score (N=3,071)		P-value
	#	COL%	#	COL%	#	COL%	
**Age at diagnosis ≥ 51**	4363	78.0	1905	75.5	2458	80.0	<.0001
**Diagnosis year ≥ 2017**	3442	61.5	1303	51.6	2139	69.7	<.0001
**Race**							0.6268
Missing (Hispanic/Other)	855	15.3	361	14.3	494	16.1	
White	3317	59.3	1506	59.7	1811	59.0	
Black/AA	1423	25.4	657	26.0	766	24.9	
**Has positive nodes**							<.0001
Missing	296	5.3	261	10.3	35	1.1	
No	3798	67.9	1214	48.1	2584	84.1	
Yes	1501	26.8	1049	41.6	452	14.7	
**Tumor Size**	N = 3268		N = 1426		N = 1842		<.0001
Mean±SD	22.4±17.2		27.2±22.1		18.7±10.6		
Median (25th, 75th)	18.0 (12.0, 26.0)		21.0 (12.0, 34.0)		16.0 (12.0, 23.0)		
**Surgery type**							<.0001
Lumpectomy	2921	52.2	1089	43.1	1832	59.7	
Mastectomy	1917	34.3	1013	40.1	904	29.4	
None	187	3.3	179	7.1	8	0.3	
Other	570	10.2	243	9.6	327	10.6	
**Stage Category**							<.0001
Missing	10	0.2	7	0.3	3	0.1	
0,1	3935	70.3	1333	52.8	2602	84.7	
2,3	1369	24.5	939	37.2	430	14.0	
4,5,7	281	5.0	245	9.7	36	1.2	
**Chemotherapy/Immunotherapy received**	1792	32.0	1250	49.5	542	17.6	<.0001
**Categorical flag for recurrence/never disease free**							<.0001
NDF & no recurrence	153	2.7	85	3.4	68	2.2	
NDF & recurrence	296	5.3	252	10.0	44	1.4	
no recurrence	3749	67.0	1246	49.4	2503	81.5	
recurrence	1397	25.0	941	37.3	456	14.8	
**Menopause age > 55**	3715	66.4	1622	64.3	2093	68.2	0.0022

**Charlson Comorbidity Index**							
**0**	3746	67.0	1670	66.2	2076	67.6	0.0674
**1,2**	1492	26.7	672	26.6	820	26.7	
**3+**	357	6.4	182	7.2	175	5.7	
**Insurance**							
**Medicaid**	133	2.4	60	2.4	73	2.4	0.454
**Medicare**	2057	36.8	909	36.0	1148	37.4	
**Commercial**	3290	58.8	1511	59.9	1779	57.9	
**Private or Self**	108	1.9	41	1.6	67	2.2	
**Other**	1	0.0	.	.	1	0.0	
**Unknown**	6	0.1	3	0.1	3	0.1	
**High deductible**	370	6.6	156	6.2	214	7.0	0.2381
**RURAL**							
**Missing**	27	0.5	11	0.4	16	0.5	0.0386
**No**	5535	98.9	2504	99.2	3031	98.7	
**Yes**	33	0.6	9	0.4	24	0.8	
**Neighborhood Deprivation Index**	N = 5580		N = 2517		N = 3063		0.0041
**Mean±SD**	-0.27±0.84		-0.24±0.84		-0.29±0.84		
**Median (25th, 75th)**	-0.38 (-0.83, 0.19)		-0.34 (-0.81, 0.22)		-0.40 (-0.84, 0.16)		

Table S2. Mortality Univariate Regression: All Variables.

Variables	Hazard Ratio	95% Confidence Limits		P-value
**Age at dx ≥ 51**	4.44	2.18	9.05	<.0001
**Year of dx ≥ 2017**	1.79	1.20	2.66	0.0040
**Race/Ethnicity**				
**Black/AA vs NHW**	1.56	1.13	2.15	0.0073
**Average BMI (N=2,570/D=160)**	1.01	0.99	1.03	0.3615
**Tumor size by 10 (N=1,594/D=93)**	1.27	1.09	1.48	0.0024
**Has positive nodes (N=2,548/D=156)**	1.35	0.86	2.10	0.1888
**Type of Surgery**				
**Mastectomy**	1.21	0.87	1.70	0.2643
**Lumpectomy**	Ref	Ref	Ref	
**Other**	0.00	0.00	0.00	0.9828
**No surgery**	0.69	0.39	1.23	0.2109
**Stage (N=2,574/D=150)**				

**0, 1**	Ref	Ref	Ref	
**2, 3**	1.26	0.79	2.02	0.3365
**4, 5, 7**	4.63	1.71	12.55	0.0026
**Chemotherapy/Immunotherapy received**	0.94	0.62	1.43	0.7801
**Days from surgery to endocrine therapy**	1.00	1.00	1.00	0.0075
**Recurrence**				
**Yes vs No**	3.66	2.66	5.03	<.0001
**High-deductible health plan**	1.00	0.44	2.26	0.9958
**Age of menopause > 55**	2.87	1.81	4.54	<.0001
**Charlson Comorbidity index**				
**0**	Ref	Ref	Ref	
**2-Jan**	1.95	1.39	2.75	0.0001
**3+**	4.94	3.19	7.66	<.0001
**Rural (N=2,565/D=159)**				
**Yes vs No**	0.63	0.09	4.53	0.6503
**Insurance**				
**Medicaid**	3.29	1.02	10.64	0.0462
**Medicare**	3.29	2.34	4.64	<.0001
**Commercial**	Ref	Ref	Ref	
**Private or Self**	0.53	0.07	3.86	0.5323
**NDI ACS 2020 (N=2,570/D=160)**	1.20	1.00	1.43	0.0447
**Oncotype Dx Score -**				
**0 - 16**	0.98	0.64	1.51	0.9411
**17 - 20**	Ref	Ref	Ref	
**21 - 25**	0.91	0.51	1.65	0.7626
**26 - 100**	1.85	1.15	2.98	0.0117
**Interaction**				
**Black vs Non-Hispanic White*Oncotype Dx Score**				0.7674

Table S3. Breast Cancer Recurrence Univariate Regression: All Variables.

Variables	Hazard Ratio	95% Confidence Limits		P-value
**Age at dx ≥ 51**	0.99	0.77	1.29	0.9652

**Year of dx ≥ 2017**	1.48	1.18	1.84	0.0006
**Average BMI**	1.00	0.99	1.02	0.8376
**Tumor size by 10 (N=1,569/R=259/D=55)**	1.40	1.31	1.49	<0.0001
**Has positive nodes (N=2,464/R=388/D=95)**	24.80	19.95	30.82	<0.0001
**Type of Surgery (N=2,487/R=392/D=98)**				
**Mastectomy**	1.77	1.43	2.19	<0.0001
**Lumpectomy**	Ref	Ref	Ref	
**Other**	1.48	1.08	2.02	0.0150
**Stage (N=2,489/R=392/D=98)**				
**0, 1**	Ref	Ref	Ref	
**2, 3**	20.79	16.74	25.80	<0.0001
**4, 5, 7**	22.54	12.98	39.13	<0.0001
**Chemotherapy/Immunotherapy received**	2.20	1.76	2.74	<0.0001
**Days from surgery to endocrine therapy (N=2,205/R=342/D=91)**	1.00	1.00	1.00	<0.0001
**Age of menopause > 55**	1.01	0.82	1.26	0.9128
**Charlson Comorbidity index**				
**0**	Ref	Ref	Ref	
**2-Jan**	1.18	0.95	1.46	0.1387
**3+**	1.11	0.74	1.68	0.6107
**Rural (N=2,481/R=390/D=98)**				
**Yes vs No**	1.11	0.42	2.97	0.8352
**Insurance (N=2,489/R=392/D=98)**				
**Medicaid**	1.73	0.92	3.25	0.0904
**Medicare**	1.23	1.00	1.50	0.0494
**Commercial**	Ref	Ref	Ref	
**Private or Self**	1.10	0.51	2.34	0.8148
**High deductible**	0.71	0.42	1.18	0.1867
**NDI ACS 2020 (N=2,486/R=392/D=98)**	0.96	0.85	1.08	0.5042
**Race/Ethnicity**				
**Black/AA vs NHW**	0.90	0.72	1.13	0.3681
**Oncotype Dx Score -**				
**0 - 16**	0.98	0.75	1.28	0.8842
**17 - 20**	Ref	Ref	Ref	
**21 - 25**	0.79	0.53	1.15	0.2172
**26 - 100**	1.61	1.19	2.18	0.0019

Interaction				
Black vs NHW*Oncotype Dx Score				0.0696

Table S4. Patient Population by Black/African American Race limited to 0–16 Oncotype Dx score.

---------------	All		Race				P-value
			NHW		Black/AA		
	N = 1,323		N = 997		N = 326		
	N	COL%	N	COL%	N	COL%	
**Age at dx ≥ 51**							0.0050
**No**	247	18.7	169	17	78	23.9	
**Yes**	1076	81.3	828	83	248	76.1	
**Year of dx ≥ 2017**							0.0006
**No**	423	32	344	34.5	79	24.2	
**Yes**	900	68	653	65.5	247	75.8	
**Has positive nodes**							0.5366
**Missing**	18	1.4	14	1.4	4	1.2	
**No**	1092	82.5	819	82.1	273	83.7	
**Yes**	213	16.1	164	16.4	49	15	
**Average BMI**	N = 1319		N = 994		N = 325		<0.0001
**Mean±SD**	31.0±7.2		30.3±7.0		33.2±7.2		
**Median (25th, 75th)**	30.1 (25.6, 35.7)		29.3 (25.1, 34.8)		32.5 (27.7, 37.7)		
**Tumor size**	N = 835		N = 731		N = 104		0.1405
**Mean±SD**	18.3±10.5		18.5±10.6		16.7±10.3		
**Median (25th, 75th)**	16 (12, 22)		16 (12, 22)		15 (10, 19)		
**Type of Surgery**							0.0392
**Lumpectomy**	792	59.9	594	59.6	198	60.7	
**Mastectomy**	374	28.3	271	27.2	103	31.6	
**None**	4	0.3	3	0.3	1	0.3	
**Other**	153	11.6	129	12.9	24	7.4	
**Stage**							0.4198
**Missing**	2	0.2	1	0.1	1	0.3	
**0, 1**	1109	83.8	834	83.7	275	84.4	
**2, 3**	200	15.1	151	15.1	49	15	
**4, 5, 7**	12	0.9	11	1.1	1	0.3	
**Chemotherapy/Immunotherapy received**							0.1423
**No**	1265	95.6	958	96.1	307	94.2	
**Yes**	58	4.4	39	3.9	19	5.8	
**Endocrine therapy received**							0.0826
**No**	153	11.6	124	12.4	29	8.9	

**Yes**	1170	88.4	873	87.6	297	91.1	
**Time from surgery to endocrine therapy, in days**	N = 1155		N = 859		N = 296		0.6881
**Mean±SD**	91±71		93±66		83±83		
**Median (25th, 75th)**	89 (42, 121)		92 (48, 122)		77 (34, 112)		
**Recurrence**							0.0160
**Never disease free & no recurrence**	26	2	14	1.4	12	3.7	
**Never disease free & recurrence**	17	1.3	12	1.2	5	1.5	
**No recurrence**	1093	82.6	819	82.1	274	84	
**Recurrence**	187	14.1	152	15.2	35	10.7	
**Age of menopause > 55**							0.0109
**No**	385	29.1	272	27.3	113	34.7	
**Yes**	938	70.9	725	72.7	213	65.3	
**Charlson Comorbidity Index**							<0.0001
**0**	897	67.8	706	70.8	191	58.6	
**1-2**	345	26.1	245	24.6	100	30.7	
**3+**	81	6.1	46	4.6	35	10.7	
**Insurance**							0.0010
**Medicaid**	28	2.1	16	1.6	12	3.7	
**Medicare**	541	40.9	423	42.4	118	36.2	
**Commercial**	726	54.9	532	53.4	194	59.5	
**Private or Self**	27	2	26	2.6	1	0.3	
**Unknown**	1	0.1	.	.	1	0.3	
**High deductible**							0.1949
**No**	1247	94.3	935	93.8	312	95.7	
**Yes**	76	5.7	62	6.2	14	4.3	
**RURAL**							0.0318
**Missing**	8	0.6	5	0.5	3	0.9	
**No**	1301	98.3	978	98.1	323	99.1	
**Yes**	14	1.1	14	1.4	.	.	
**Neighborhood Deprivation Index ACS 2020**	N = 1319		N = 994		N = 325		<0.0001
**Mean±SD**	0.25±0.80		0.44±0.69		0.18±0.92		
**Median (25th, 75th)**	0.40 (0.81,0.10)		0.48 (0.88,0.09)		0.03 (0.53,0.63)		
